# Tumour-free distance: a novel prognostic marker in patients with early-stage cervical cancer treated by primary surgery

**DOI:** 10.1038/s41416-020-01204-w

**Published:** 2020-12-14

**Authors:** David Cibula, Jiri Slama, Lukáš Dostálek, Daniela Fischerová, Anna Germanova, Filip Frühauf, Pavel Dundr, Kristyna Nemejcova, Jiri Jarkovsky, Silvie Sebestova, Andrea Burgetová, Martina Borčinová, Roman Kocián

**Affiliations:** 1 Gynecologic Oncology Center, Department of Obstetrics and Gynecology, First Faculty of Medicine, Charles University in Prague and General University Hospital in Prague, Apolinarska 18, Prague 2, 12800 Czech Republic; 2 Department of Pathology, First Faculty of Medicine, Charles University in Prague and General University Hospital in Prague, Studnickova 2, Prague 2, 12800 Czech Republic; 3 Institute of Biostatistics and Analyses, Faculty of Medicine, Masaryk University, Kamenice 126/3, Brno, 62500 Czech Republic; 4 Institute of Health Information and Statistics of the Czech Republic, Palackeho Namesti 4, P.O. Box 60, Prague 2, 12801 Czech Republic; 5 Department of Radiology, First Faculty of Medicine, Charles University in Prague and General University Hospital in Prague, U Nemocnice 499/2, Prague 2, 12808 Czech Republic

**Keywords:** Surgical oncology, Cervical cancer

## Abstract

**Background:**

Models predicting recurrence risk (RR) of cervical cancer are used to tailor adjuvant treatment after radical surgery. The goal of our study was to compare available prognostic factors and to develop a prognostic model that would be easy to standardise and use in routine clinical practice.

**Methods:**

All consecutive patients with early-stage cervical cancer treated by primary surgery in a single referral centre (01/2007–12/2016) were eligible if assessed by standardised protocols for pre-operative imaging and pathology. Fifteen prognostic markers were evaluated in 379 patients, out of which 320 lymph node (LN)-negative.

**Results:**

The best predictive model for the whole cohort entailed a combination of tumour-free distance (TFD) ≤ 3.5 mm and LN positivity, which separated two subgroups with a substantially distinct RR 36% and 6.5%, respectively. In LN-negative patients, a combination of TFD ≤ 3.5 mm and adenosquamous tumour type separated a group of nine patients with RR 33% from the rest of the group with 6% RR.

**Conclusions:**

A newly identified prognostic marker, TFD, surpassed all traditional tumour-related markers in the RR assessment. Predictive models combining TFD, which can be easily accessed on pre-operative imaging, with LN status or tumour type can be used in daily practice and can help to identify patients with the highest RR.

## Background

Prognostic markers in early-stage cervical cancer are used to tailor the type of surgery, including lymph node (LN) staging, type of parametrectomy and, most importantly, the administration of adjuvant treatment.^1,2^ The multiple studies that have analysed prognostic markers in these patients in the last two decades differed substantially in numerous aspects, such as cohort size, source of the data, duration of the study interval, design, selection of evaluated markers and method of statistical analysis.^3–19^ The main limitation in the majority of studies was an insufficient standardisation of both clinical management during the study period, and, even more importantly, the method of assessment of individual markers. It is not surprising that the outcomes of these studies were inconsistent. Only a few prognostic markers were identified unanimously, including tumour size, stage of disease and LN involvement. Discrepant results were found for many other markers, such as age, lymphovascular space invasion (LVSI), depth of stromal invasion (DSI) or grade.

In an effort to avoid these limitations, we assessed the majority of traditional and a few rarely evaluated clinical and pathological variables in a large cohort of patients enrolled in a single institutional database. All cases were assessed by a standardised protocol for both pre-operative imaging and pathology. The goal of this study was to compare individual markers and identify those with the highest significance for the risk of recurrence and to develop a simple prognostic model, which would be easy to standardise and use in routine practice. An ideal model should differentiate subgroups of patients with the most profoundly different recurrence rate (RR).

## Methods

### Patient selection

**Fig. 1:**
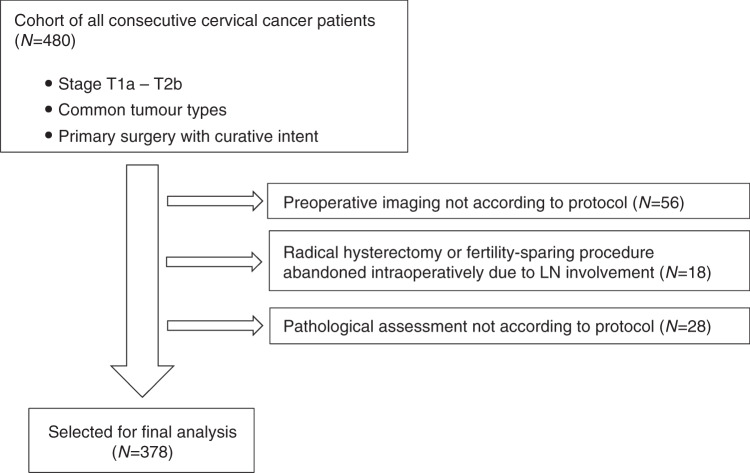
Study flow chart.

All consecutive patients with early-stage cervical cancer (stages T1a–T2b) who were treated by primary surgery with curative intent in a single tertiary gynaecologic oncology centre from January 2007 to December 2016 were enrolled in the study. The main inclusion criteria consisted of: (a) histologically proven cervical cancer; (b) common tumour types: squamous cell carcinoma, adenocarcinoma or adenosquamous carcinoma; (c) stage T1a–T2b; (d) LNs not enlarged or suspicious on pre-operative imaging; (e) primary surgical treatment with curative intent. Patients in whom radical hysterectomy or fertility-sparing procedure was abandoned intraoperatively and in whom pre-operative imaging or pathological assessment was not performed according to standardised protocols were excluded (Fig. 1).

The study protocol was approved by the Institutional Review Board on 10/2017 under the registration number 1587/17 S-IV. Due to the retrospective nature of the study, a need for informed consent was waived.

### Patient management

Surgical management has not been substantially changed within the time span of the study. Open surgery was the prevailing approach for radical hysterectomy or radical trachelectomy. The first step of the management was LN intraoperative assessment. SLN biopsy was performed selectively in the early period, and, it has become a standard part of the surgical management in our institution since 2010. If positive pelvic LN/SLN was detected during the surgery, cervical/uterine procedure was abandoned, and the patient was referred for primary chemoradiation (these cases were excluded from this analysis). In the majority of the patients, SLN biopsy was followed by a systematic pelvic lymphadenectomy (PLND), except for stage T1a/LVSI negative or in patients enrolled into a prospective SENTIX trial. The technique of systematic PLND remained unchanged and included seven anatomical regions in the pelvis, as previously described.^20^ The Querleu–Morrow classification was used for the description of the type of parametrectomy.^21^ The radicality of parametrectomy was tailored according to cervical prognostic risk factors. In stage T1a, a simple hysterectomy or conisation was performed; in higher stages, radical hysterectomy type C1 (nerve-sparing) was indicated in patients with smaller tumours, defined as a tumour-free distance >0 mm (TFD, defined in “Pre-operative imaging” section) and the tumour size ≤3 cm, while type C2 was indicated in larger tumours and in those with no remaining TFD on either side of the cervix. Patients with LN involvement from the final pathology, patients with positive surgical margins or patients with parametrial invasion were referred to adjuvant treatment.

All cases were followed in the institution for at least 5 years after the treatment. Follow-up visits were scheduled every 3 months in the first 2 years, and in 6–12 months intervals thereafter, according to the presence of negative prognostic factors. One of the imaging tests (expert ultrasound or CT or PET/CT) was performed in all patients between 6 and 12 months interval after the surgery or after the adjuvant treatment has been finished. Further imaging tests were done only if clinically indicated, either in the presence of symptoms or any suspicious finding on physical examination. The diagnosis of recurrence was defined as (a) unequivocal finding on imaging; (b) suspicious recurrence on imaging either confirmed by biopsy or supported by other signs (disease progression on imaging or progression of symptoms) or death caused by disease or death of unknown cause. The outcome of patients was matched with the Czech National Database of Death Certificates, so mortality data and a cause of death were verified.

### Pre-operative imaging

In the first period, 2000–2006, the combination of magnetic resonance imaging (MRI) and expert ultrasound (US) was routinely used.^22^ Given better results of US in the local assessment, patients were examined only by US during the following period.^22–24^

US images were obtained with a GE Logic 9 and Voluson E8 ultrasound machine (GE Medical Systems, Milwaukee, WI, USA) equipped with an endoluminal microconvex linear array probe of 5–9 MHz frequency and a transabdominal convex linear array probe of 5 MHz frequency. Patients were examined in the lithotomy position with an empty bladder. Transrectal placement of the probe was preferred to transvaginal approach, not only due to the lower risk of tumour bleeding but the evaluation of the distal part of the cervix is often less hampered by artefacts (acoustic shadows) and it allows for a closer distance between the probe and the tumour.^25^

**Fig. 2:**
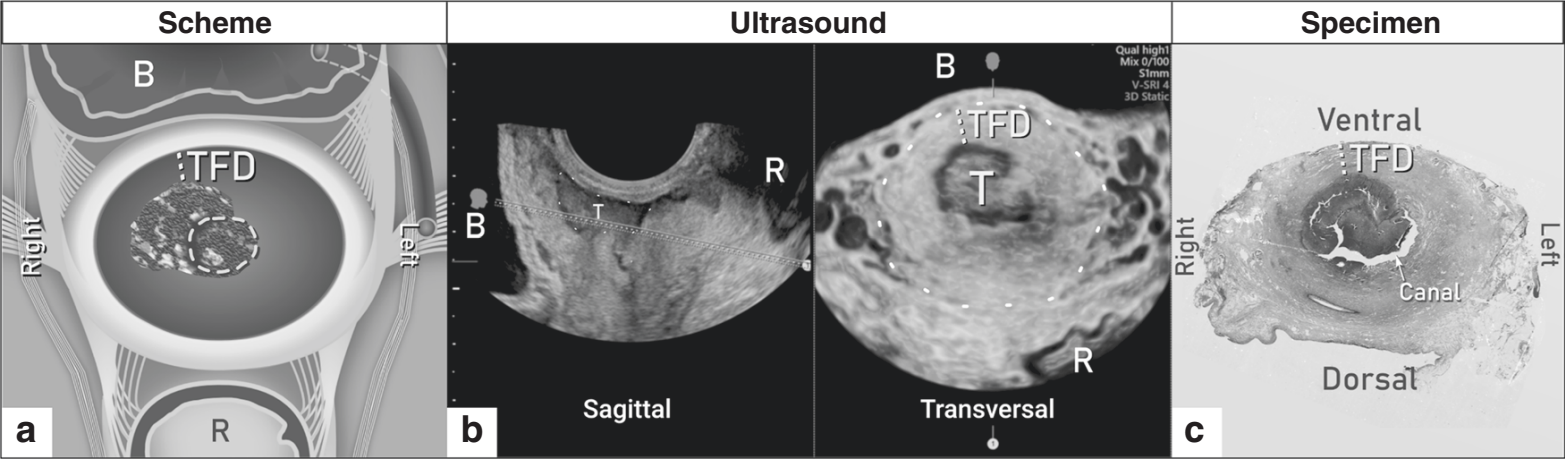
Tumour-free distance (TFD) assessment. **a** Scheme of TFD measurement as the minimal lateral distance of uninvolved stroma between the tumour and pericervical ring; **b** TFD measurement by ultrasound in a transversal plane; **c** TFD measurement on pathological specimen.

All patients underwent a standardised US examination by an experienced examiner (level 3 according to the recommendations for the practice of the medical US of the European Federation of Societies for Ultrasound in Medicine and Biology);^26^ the methodology was described in more detail elsewhere.^27^ Using the real-time 2D-US (greyscale and power Doppler), the examiner followed a standardised protocol and evaluated the presence of the tumour, the tumour size in three diameters, the distance between the cranial pole of the tumour and the internal cervical os, the integrity of the pericervical ring, the involvement of parametria and adjacent organs and the retroperitoneal LNs (Supplementary Table S1). TFD was measured on a transversal plane as a minimum distance of uninvolved stroma between the tumour and pericervical ring (dense hyperechogenic layer surrounding the cervix) (Fig. 2). If no residual tumour was found by imaging after conisation, TFD was calculated as half of the cervical transversal diameter. A standardised US protocol was completed in the web-based central database, data were locked after an examination, and the database did not allow any subsequent changes. When clinically indicated, or if any limitation of US was encountered, the appropriate complementary imaging method was completed and reviewed by an experienced radiologist dedicated to gynaecologic oncology.

### Pathological assessment

For the frozen section, every SLN/ LN was bisected, and each half was examined. Each SLN was processed as a whole by cutting 2-mm-thick slices perpendicular to its long axis and analysed using ultrastaging protocol, as described previously.^28^ Non-SLNs were processed completely, in 2-mm-thick slices. LNs smaller than 3 mm were not sectioned.

Processing of the hysterectomy specimen consisted of macroscopic description, grossing, sampling and histological examination which were carried out in accordance with a standardised institutional protocol reflecting international and national guidelines. Each biopsy report was accompanied by a checklist summarising all relevant markers, such as tumour size in three diameters, tumour location, relationship to surrounding structures and presence of LVSI. A detailed description of the hysterectomy specimen processing can be found in Supplementary Table S2.

### Prognostic markers

The method used for the assessment of individual markers is reported in the article as “P” for pathology or “I” for imaging. Together, 15 prognostic markers were evaluated, including age, 11 tumour-related and three LN status-related ones (LN positivity, number of positive LNs; type of metastasis in LN). Amongst tumour-related factors, seven were related to tumour size assessment: stage (P), largest tumour size (P and I), tumour volume calculated by the formula for ellipsoid from pathological measurement (P), largest tumour size binarised (P), depth of stromal invasion (DSI) (P), minimal TFD (I) defined as the minimal uninvolved stroma between the tumour and pericervical ring (dense hyperechogenic layer on ultrasound while hypointense layer on MRI) on either side of the cervix, minimal TFD binarised (I); and four markers related to pathology or local disease spread: LVSI, tumour type, grade, parametrial invasion (P). Gynaecologic Oncology Group score (GOG score) was calculated according to the GOG criteria combining LVSI status, DSI and tumour size.^3^

### Statistical analysis

Three groups of patients were analysed separately: (a) all patients enrolled in the study; (b) patients without LN involvement (after excluding cases with micrometastases, micrometastases, and isolated tumour cells); (c) patients who did not receive adjuvant treatment.

Standard descriptive statistics were applied in the analysis; absolute and relative frequencies for categorical variables and median supplemented with the 5th–95th percentile range for continuous variables. The influence of patients’ characteristics on their recurrence risk was analysed using univariate and multivariate Cox proportional hazards models and described using hazard ratios (HRs) and their 95% confidence intervals. The multivariate model was computed using a forward stepwise algorithm on a subset of predictors statistically significant in univariate analysis. Cut-off values for continuous variables were determined by ROC analysis; the criterion was the highest value of the sum of sensitivity and specificity. AUC from ROC analysis for multivariate models was adopted as a measure of their overall predictive power. Kaplan–Meier methodology was adopted for the visualisation of recurrence-free survival (RFS) data according to patient categories derived from variable combinations in multivariate models; the statistical significance of RFS curve differences among groups of patients was tested using the log-rank test. The analysis was computed using SPSS 25.0.0.1 (IBM Corporation 2018).

## Results

### Group characteristics

Table 1 presents the characteristics of two cohorts, the whole group (*N* = 379) and the LN-negative group (*N* = 320) after all cases with any type of metastasis (macrometastases (MAC), micrometastases (MIC) and isolated tumour cells (ITC)) were excluded.

**Table 1:** Summary of demographic and clinical parameters in the whole cohort and in lymph node (LN)-negative patients.

		All patients^a^, *N* = 379 (Cohort A)	LN-negative patients^a^ (without MAC, MIC, ITC), *N* = 320 (Cohort B)
Age (years)		41.9 (27.8; 70.3)	41.8 (27.7; 70.7)
BMI		24.4 (18.4; 36.6)	24.7 (18.7; 36.1)
Stage (pT)	1a1	66 (17.4%)	66 (20.6%)
	1a2	9 (2.4%)	9 (2.8%)
	1b1	203 (53.6%)	182 (56.9%)
	1b2	46 (12.1%)	27 (8.4%)
	2a	11 (2.9%)	7 (2.2%)
	2b	44 (11.6%)	29 (9.1%)
Tumour type	Adenocarcinoma	76 (20.1%)	63 (19.7%)
	Adenosquamous	11 (2.9%)	6 (1.9%)
	Squamous	287 (75.7%)	247 (77.1%)
	Missing	5 (1.3%)	4 (1.3%)
Grade	1	44 (11.6%)	44 (13.7%)
	2	171 (45.1%)	150 (46.9%)
	3	164 (43.3%)	126 (39.4%)
LVSI		144 (38.0%)	97 (30.3%)
Fertility-sparing treatment		65 (17.2%)	62 (19.4%)
Type of parametrectomy	SH or C*	51 (13.5%)	51 (16.0%)
	B	33 (8.6%)	29 (9.0%)
	C1	133 (35.1%)	119 (37.3%)
	C2	162(42.8%)	121 (37.7%)
SLN biopsy		234 (61.7%)	194 (60.6%)
Pelvic lymphadenectomy		301 (79.4%)	244 (76.3%)
Number of removed LN per patient		31.0 (0.0; 58.0)	30.5 (0.0; 58.0)
Type of LN metastases	MAC	32 (8.4%)	–
	MIC	18 (4.7%)	–
	ITC	9 (2.4%)	–
	Negative	320 (84.4%)	320 (100.0%)
Pre-operative assessment by imaging: largest tumour size (mm)		22.0 (0.8; 54.0)	19.0 (0.0; 52.0)
Minimal TFD (mm)		3.0 (0.0; 14.0)	4.0 (0.0; 14.0)
Pathological assessment: largest tumour size (mm)		24.0 (2.5; 65.0)	20.0 (2.2; 57.0)
Depth of stromal invasion (mm)		15.0 (5.0; 25.6)	14.0 (5.0; 25.0)
Tumour volume (mm^3^)		3811.8 (7.3; 44,588.8)	2 358.8 (4.2; 39,964.5)
Adjuvant treatment		75 (20.1%)	33 (10.3%)
Follow-up length (months)		78.1 (9.2; 152.8)	78.4 (9.3; 152.8)
Recurrence rate		43 (11.3%)	23 (7.2%)

*ITC* isolated tumour cells, *LVSI* lymphovascular space invasion, *MAC* macrometastasis, *MIC* micrometastasis.

^a^Absolute and relative frequencies for categorical variables; median supplemented with 5^th^–95^th^ percentile range for continuous variables; *simple hysterectomy or conisation.

LN involvement was detected in 59 cases (16%), from which 8.4% were MAC, 4.7% MIC and 2.4% ITC. The surgical approach was mostly open surgery. Only 22 (7%) radical hysterectomies were performed by laparoscopy. Sentinel lymph node (SLN) biopsy was not performed in 145 cases (38%): due to the failure of detecting SLN (*N* *=* 30), early disease stages which did not require LN assessment (stage T1a1 or T1a2/ LVSI neg) (*N* *=* 59), cases prior to 2009 (*N* *=* 32) when SLN started to be performed routinely, and other reasons (*N* *=* 24). Pelvic lymph node dissection (PLND) was not performed in 78 cases (21%): 69 of them were in stage T1a with either no LN staging or just SLN biopsy, and 9 patients after 2016 were enrolled into the prospective SENTIX trial, in which part of the management is SLN biopsy only, without PLND. In the whole cohort, 75 patients (20%) were referred to adjuvant treatment for the following reasons: positive parametria (11), positive vaginal margins (2), positive LN (59), other reasons (3) (for details see Supplementary Table S3). The RR reached 11.3% in the whole group and 7.2% in the LN-negative cohort with a median follow-up of 78 months in both groups.

### Univariate and multivariate analyses of prognostic markers

Fifteen markers were evaluated in univariate analysis for predicting RR (Table 2). The highest hazard ratio (HR) was found for adenosquamous tumour type (HR 7.29 (3.12; 17.01)), stage ≥1b2 (HR 5.99 (1.69; 21.24)), LN involvement (HR 5.46 (2.99; 9.95)), tumour size ≥32 mm (HR 3.69 (2.01; 6.78)), and tumour-free distance (TFD) ≤ 3.5 mm (HR 7.16 (2.52; 20.39)). TFD cut-off was determined by ROC analysis, the criterion was the highest value of the sum of sensitivity and specificity. All markers related to the tumour size were significant (largest tumour size assessed by pathology (P), tumour volume (P), minimal TFD assessed by imaging (I), DSI (P) and tumour volume (P)). Only four markers were significant in the cohort of LN-negative patients (adenosquamous tumour type, grade 2, minimal TFD, TFD ≤ 3.5 mm).

**Table 2:** Univariate analysis of recurrence predictors.

		All patients (Cohort A)			LN-negative patients (without MAC, MIC, ITC) (Cohort B)		
Predictor^a^		Total *N* (relapse/event)	HR (95% CI)	*P* value^d^	Total *N* (relapse/event)	HR (95% CI)	*P* value^d^
Age (years)		379 (43)	1.00(0.98; 1.03)	0.725	320 (23)	0.98 (0.95; 1.02)	0.345
Stage pT	1a	75 (3)	Ref.		75 (3)	Ref.	
	1b1–1b2	249 (28)	3.39 (1.03; 11.16)	**0.045**	209 (15)	2.13 (0.61; 7.35)	0.234
	>1b2	55 (12)	5.99 (1.69; 21.24)	**0.006**	36 (5)	3.67 (0.88; 15.35)	0.075
Tumour type	Squamous	280 (26)	Ref.		241 (13)	Ref.	
	Adenocarcinoma	76 (10)	1.53 (0.74; 3.17)	0.255	63 (7)	2.35 (0.94; 5.90)	0.069
	Adenosquamous	18 (7)	7.29 (3.12; 17.01)	**<0.001**	12 (3)	8.32 (2.32; 29.86)	**0.001**
Grade	1	37 (6)	ref.		36 (6)	ref.	
	2	144 (13)	0.57 (0.22; 1.51)	0.257	122 (6)	0.31 (0.10; 0.95)	**0.040**
	3	138 (23)	1.10 (0.45; 2.69)	0.841	103 (11)	0.66 (0.24; 1.77)	0.406
LVSI	No	235 (18)	Ref.		223 (17)	Ref.	
	Yes	144 (25)	2.48 (1.35; 4.54)	**0.003**	97 (6)	0.84 (0.33; 2.12)	0.706
Number of positive LN		379 (43)	1.18 (1.11; 1.26)	**<0.001**	320 (23)	–	–
LN positivity	No (any type)	319 (23)	Ref.		320 (23)	–	–
	Yes	60 (20)	5.46 (2.99; 9.95)	**<0.001**	0 (0)	–	–
LN positivity	ITC, negative	329 (25)	ref.		320 (23)	–	–
	MAC, MIC	50 (18)	5.40 (2.94; 9.90)	**<0.001**	0 (0)	–	–
Largest tumour size (P)		378 (43)	1.02 (1.00; 1.03)	**0.007**	319 (23)	1.00 (0.98; 1.02)	0.909
Largest tumour size (P) binarised^b^	≤Cut-off^c^	263 (18)	Ref.		195 (12)	Ref.	
	>Cut-off^c^	115 (25)	3.69 (2.01; 6.78)	**<0.001**	124 (11)	1.54 (0.68; 3.50)	0.299
Depth of stromal invasion (P)		378 (27)	1.06 (1.00; 1.12)	**0.035**	187 (12)	1.04 (0.95; 1.14)	0.379
Largest tumour size (I)		379 (43)	1.03 (1.01; 1.05)	**<0.001**	320 (23)	1.01 (0.99; 1.04)	0.265
Parametrial invasion (P)	No	323 (33)	Ref.		281 (19)	Ref.	
	Yes	56 (10)	1.70 (0.84; 3.45)	0.141	39 (4)	1.47 (0.50; 4.31)	0.488
Minimal TFD (I)		321 (33)	0.84 (0.76; 0.93)	**0.001**	278 (18)	0.89 (0.79; 0.99)	**0.030**
TFD binarised (I)^b^	>3.5	146 (4)	Ref.		140 (4)	Ref.	
	≤3.5	175 (29)	7.16 (2.52; 20.39)	**<0.001**	134 (14)	4.37 (1.44; 13.27)	**0.009**
Tumour volume (mm^3^)		361 (43)	1.00 (1.00; 1.00)	**0.021**	302 (23)	1.00 (1.00; 1.00)	0.831

*CI* confidence interval, *ITC* isolated tumour cells, *LN* lymph node, *MAC* macrometastasis, *MIC* micrometastasis, *TFD* tumour-free distance, *(I)* assessed by imaging, *(P)* assessed by pathology.

^a^Hazard ratios are computed using Cox proportional hazards model; ^b^cut-off determined by ROC analysis, the criterion was the highest value of the sum of sensitivity and specificity; ^c^cut-off for Cohort A: 32.5, Cohort B: 25.5; ^d^level of significance *P* < 0.05.

**Fig. 3:**
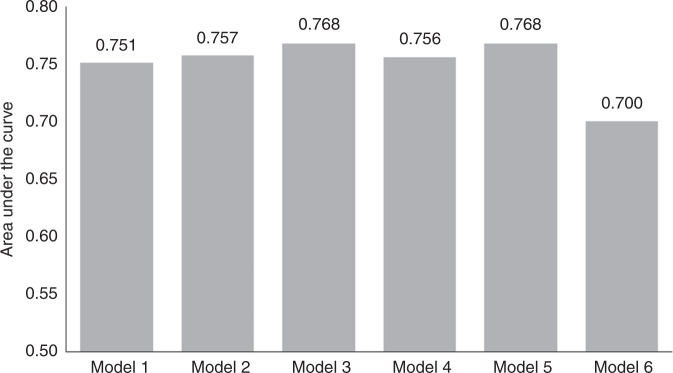
Accuracy of prognostic models for recurrence risk in the whole cohort (ROC analysis). Model 1: Minimal tumour-free distance (TFD), number of positive lymph nodes (LN); Model 2: TFD binarised, LN positivity binarised; Model 3: TFD binarised, LN positivity binarised, lymphovascular space invasion; Model 4: TFD binarised, LN positivity binarised, depth of stromal invasion; Model 5: TFD binarised, LN positivity binarised, tumour type; Model 6: largest tumour size binarised, LN positivity binarised.

All significant markers related to RR in univariate analyses were included in multivariate analyses using the Cox proportional hazards model (Table 3). Comparison of models according to AUC values is shown in Fig. 3. There were no major differences between Models 1 and 5 (AUC between 0.751 and 0.768). Model 2 was selected as the simplest, composed of two factors only, both in binary format: LN positivity and TFD *≤* 3.5 mm. Combining the model with additional tumour-related markers, such as LVSI, DSI or tumour type, did not substantially improve the reliability of the model. Replacing TFD with the largest tumour size (>32.5 mm) substantially decreased the model’s reliability (AUC *=* 0.700).

**Table 3:** Multivariate analysis of prognostic markers in the whole group.

Whole cohort		HR (95% CI)	*P* value^b^
*Model 1*			
Minimal TFD (I)		0.85 (0.77; 0.94)	**0.002**
Number of positive LN		1.14 (1.05; 1.23)	**0.001**
*Model 2*			
TFD (I) binarised^a^	≤3.5 (ref. >3.5)	4.58 (1.52; 13.80)	**0.007**
LN positivity	Yes (ref. no)	3.79 (1.83; 7.81)	**<0.001**
*Model 3*			
TFD (I) binarised^a^	≤3.5 (ref. >3.5)	4.57 (1.50; 13.97)	**0.008**
LN positivity	Yes (ref. no)	3.76 (1.67; 8.50)	**0.001**
LVSI	Yes (ref. no)	1.01 (0.45; 2.28)	0.976
*Model 4*			
TFD (I) binarised^a^	≤3.5 (ref. >3.5)	3.48 (0.71; 17.00)	0.123
LN positivity	Yes (ref. no)	5.02 (2.26; 11.14)	**<0.001**
Stromal invasion (P)		1.00 (0.94; 1.07)	0.989
*Model 5*			
TFD (I) binarised^a^	≤3.5 (ref. >3.5)	4.13 (1.16; 14.69)	**0.029**
LN positivity	Yes (ref. no)	3.8 (1.82; 7.92)	**<0.001**
Tumour type	Adeno (ref. squamo)	1.39 (0.26; 7.49)	0.700
	Adenosquamo (ref. squamo)	1.24 (0.2; 7.78)	0.816
*Model 6*			
Largest tumour size (P) binarised^a^	>32.5 (ref. ≤32.5)	2.29 (1.16; 4.49)	**0.016**
LN positivity	Yes (ref. no)	3.78 (1.94; 7.36)	**<0.001**

*adeno* adenocarcinoma, *CI* confidence interval, *LN* lymph node, *LVSI* lymphovascular space invasion, *squamo* squamous cell cancer, *TFD* tumour-free distance, *(I)* assessed by imaging, *(P)* assessed by pathology.

^a^Cut-off determined by ROC analysis, the criterion was the highest value of the sum of sensitivity and specificity. ^b^level of significance *P* < 0.05.

**Fig. 4:**
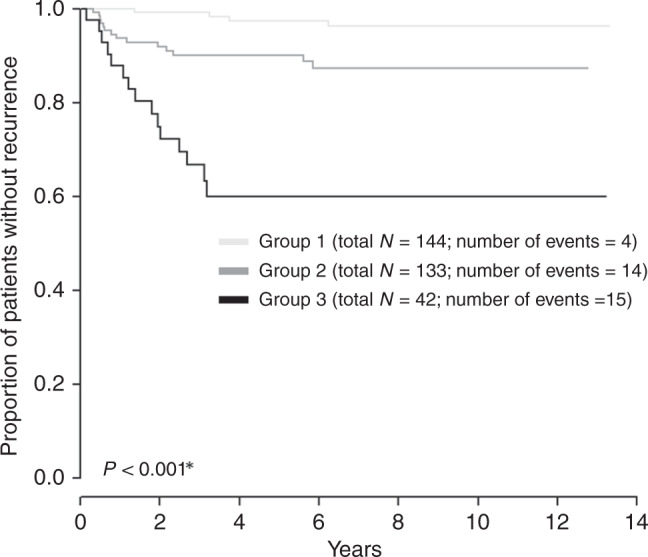
Kaplan–Meier recurrence-free survival (RFS) curve for Model 2 in the whole cohort. Group description: Group 1: tumour-free distance (TFD) > 3.5**, N0**; Group 2: TFD ≤ 3.5**, N0**; Group 3: TFD ≤ 3.5**, N1**. Two patients without event are not included in the groups: minimal tu-pcf (US) > 3.5**; a number of positive LN > 0**. *Log-rank test. **Cut-off determined by ROC analysis, the criterion was the highest value of the sum of sensitivity and specificity.

Figure 4 shows the Kaplan–Meier RFS curve for Model 2. RR in group one (N0 + TFD ≥ 3.5 mm) and two (N0 + TFD < 3.5 mm) was 2.8% and 10.5%, respectively, while in group three (TFD < 3.5 mm + N1) there were only 42 patients and the RR risk reached 36%.

**Fig. 5:**
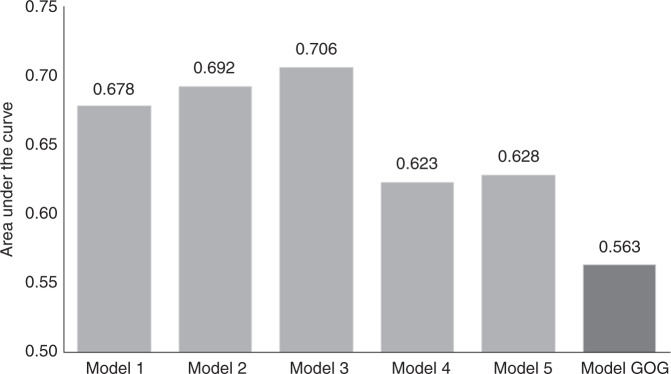
Accuracy of prognostic models for disease recurrence in lymph node (LN)-negative patients (ROC analysis). Model 1: minimal tumour-free distance (TFD), tumour type; Model 2: TFD binarised, tumour type; Model 3: TFD binarised; tumour type, lymphovascular space invasion; Model 4: TFD binarised, tumour type, depth of stromal invasion; Model 5: largest tumour size binarised, tumour type; Model 6: GOG score.

**Fig. 6:**
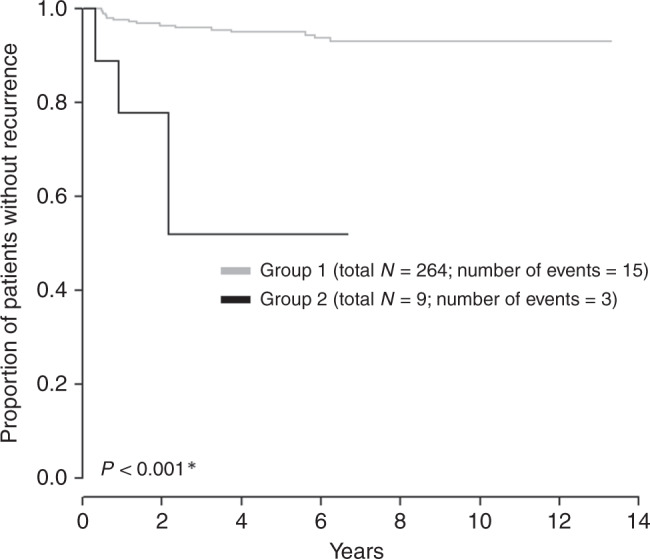
Kaplan–Meier recurrence-free survival (RFS) curve for Model 2 in lymph node-negative patients. Group description (number of patients related to this characteristic is in the bracket): Group 1: other combinations not included in Group 2; Group 2: tumour-free distance ≤3.5**, adenosquamous tumour type. One patient without event is not included into the groups: Minimal tu-pcf (US) > 3.5**, adenosquamous tumour type. *Log-rank test. **Cut-off determined by ROC analysis, the criterion was the highest value of the sum of sensitivity and specificity.

Models for LN-negative patients were composed of minimal TFD, tumour type, TFD *≤* 3.5 mm, LVSI, DSI and largest tumour size >32.5 mm (Table 4 and Fig. 5). An additional model was calculated according to the GOG 92 study.^3^ There were substantial differences in accuracy between the models (Fig. 5). Model 2 was selected as the simplest and clinically most relevant. It included only two binary factors, TFD *≤* 3.5 mm and tumour type. The model became less accurate when TFD was replaced by the largest tumour size (Model 5, Table 4). The prognostic model constructed based on GOG intermediate-risk criteria, using traditional tumour-related risk factors, such as LVSI, tumour size, and DSI, were substantially less reliable. The Kaplan–Meier curve for Model 2 is shown in Fig. 6. It separated two groups, one of them with only nine cases and a RR of 33% (TFD *≤* 3.5, adenosquamous tumour type), the other one with the majority of patients and a RR of only 6% (other combinations).

**Table 4:** Multivariate analysis of prognostic markers in LN-negative patients.

LN-negative patients		HR (95% CI)	*P* value^b^
*Model 1*			
Minimal TFD (I)		0.9 (0.81; 1.01)	0.079
Tumour type	Adeno (ref. squamo)	1.46 (0.46; 4.59)	0.521
	Adenosquamo (ref. squamo)	6.87 (1.82; 25.92)	**0.004**
*Model 2*			
TFD(I) binarised^a^	≤3.5 (ref. >3.5)	3.57 (1.15; 11.09)	**0.028**
Tumour type	Adeno (ref. squamo)	1.48 (0.47; 4.66)	0.506
	Adenosquamo (ref. spino)	6.41 (1.70; 24.22)	**0.006**
*Model 3*			
TFD (I) binarised^a^	≤3.5 (ref. >3.5)	3.93 (1.25; 12.39)	**0.020**
Tumour type	Adeno (ref. squamo)	1.47 (0.47; 4.63)	0.511
	Adenosquamo (ref. squamo)	5.83 (1.53; 22.24)	**0.010**
LVSI	Yes (ref. no)	0.60 (0.19; 1.88)	0.383
*Model 4*			
TFD (I) binarised^a^	≤3.5 (ref. >3.5)	2.90 (0.51; 16.34)	0.229
Tumour type	Adeno (ref. squamo)	1.46 (0.38; 5.58)	0.580
	Adenosquamous (ref. squamo)	2.18 (0.27; 17.95)	0.468
Stromal invasion (P)		1 (0.89; 1.13)	0.959
*Model 5*			
Largest tumour size (P) binarised^a^	>25.5 (ref. ≤25.5)	1.35 (0.58; 3.13)	0.480
Tumour type	Adeno (ref. squamo)	2.37 (0.94; 5.95)	0.067
	Adenosquamous (ref. squamo)	7.53 (2.05; 27.68)	**0.002**

*adeno* adenocarcinoma, *CI* confidence interval, *LN* lymph node, *LVSI* lymphovascular space invasion, *squamo* squamous cell cancer, *TFD* tumour-free distance, *(I)* assessed by imaging, *(P)* assessed by pathology.

^a^Cut-off determined by ROC analysis, the criterion was the highest value of the sum of sensitivity and specificity; ^b^level of significance *P* < 0.05.

In addition, a separate analysis was conducted for the third group consisting solely of the patients who did not receive adjuvant treatment. In this cohort, only 18 recurrences and 9 deaths occurred. Univariate analysis proved the significance of the same prognostic factors as for the whole cohort. Two models were selected by multivariate analysis as the simplest and clinically most relevant, both consisting of only two parameters. The first model entailed TFD ≤ 3.5 mm and LN positivity (AUC = 0.746) and the second one consisted of TFD ≤ 3.5 mm and the number of positive LNs (AUC = 0.778).

## Discussion

In our large cohort of patients assessed by standardised protocols for pre-operative imaging and pathological assessment, we evaluated 15 tumour-related or LN-related prognostic markers. Adenosquamous tumour type, LN involvement and TFD *≤* 3.5 mm were the most significant independent factors for the risk of recurrence in the whole cohort. By combining factors significant from univariate analysis, we created simple prognostic models, entailing only two factors, both in a binary format that defined a small subgroup of patients with a significantly higher RR. In the entire cohort, the combination of LN involvement and TFD *≤* 3.5 defined a group of 13% of cases with a RR of 36%, whereas, in the rest of the cohort, the RR reached only 6.5%. In the cohort of LN-negative patients, the combination of adenosquamous tumour type and TFD *≤* 3.5 defined a group of 3% of cases with a RR of 33% in comparison to a 6% RR in the rest of the cohort.

Dozens of studies have assessed prognostic factors in the past, which largely varied in size, disease stage and study interval length.^3–19^ The majority of these studies were retrospective analyses that did not define standards of care. It should be emphasised that the selection of patients, radicality of the surgery or criteria for adjuvant treatment are all aspects that can alter the oncological outcome and, therefore, also the significance of individual prognostic factors. Another potential bias is the frequently missing standardised methodology for the assessment of individual prognostic factors. Without standardised methodology, the assessment of LN status or tumour-related markers (tumour size, stromal invasion, LVSI) become unreliable, especially in multicentre studies or if the data are retrieved from national databases.

In 2009, Biewenga et al.^19^ aimed to validate 12 published prognostic models^3–14^ in an independent population of 563 patients treated for early-stage cervical cancer. They found that the great majority of published models overestimated the risk of recurrence or death from disease in their validation group, especially in the higher-risk categories. Only two models were valid for the prediction of the recurrence-free or disease-specific survival in their patient population. Five-year recurrence-free survival (RFS) for a validated group in high-risk categories oscillated in individual models between 72 and 82%, when only 9–39% of cases were assigned into high-risk groups.

Four other prognostic models were published more recently; all proposed models comprised a combination of several markers.^5–18^ In a group of 588 patients treated over 6 years, significant prognostic factors were stage, tumour grade, the ratio of positive/removed LN and number of positive LN.^15^ In a later study, a scoring system for LN-positive patients was suggested based on an analysis of 299 patients treated over 11 years, including tumour type, number of positive LN and tumour stage.^16^ Other authors used data obtained from the SEER database in 2004–2014 and tested a new marker, the log of odds between the number of removed pelvic LN and the number of negative LN.^17^ The result was a rather complicated nomogram that, in addition to markers listed above, also included age, race, marital status, tumour grade, FIGO stage, tumour type and tumour size.^17^ Recently, a Dutch group published an analysis of prognostic markers in a large multi-institutional database of 2124 cases in stages I/IIA treated within a 30-year interval.^18^ Large tumour diameter, non-squamous tumour type, LN involvement, parametrial invasion, LVSI, deep stromal invasion and also less-radical surgery were identified as independent negative prognostic variables for survival.^18^

In our study, we compared the prognostic significance of 15 markers, which were assessed by imaging or by pathology according to a standardised protocol. None of the major principles of patient management, such as the selection of patients for primary surgical treatment, tailoring of surgical radicality, or criteria for adjuvant radiotherapy have changed during the study period. The management of patients in our series was unique in a few more aspects: (1) the majority of LN-negative patients did not receive adjuvant treatment; (2) SLN biopsy was routinely performed in the majority of the cohort; (3) SLNs were processed by an intensive pathological ultrastaging which increased the detection rate of MIC and small MAC.

Adenosquamous tumour type showed the highest RR in univariate analysis. Adenocarcinoma, on the contrary, was not associated with higher RR. In accordance with the majority of previous papers, LN involvement was a significant factor, both the presence of macrometastases and micrometastases.^3–6,8,9,11^ Parametrial invasion in our study was not significant for the prognosis. Our results cannot, however, be compared to other cohorts of patients which included patients with locally advanced stages.^16–18^ Only a limited number of cases with minimal invasion into parametria according to pre-operative imaging were selectively referred to primary surgery and enrolled in our study.

A separate analysis was conducted for patients with negative LN. These patients have an excellent prognosis, which makes most of the markers insignificant given the small number of recurrences. Even in this cohort, adenosquamous tumours and TFD were identified as individual significant prognostic markers.

All markers related to the assessment of tumour size were significant in univariate analysis and the most significant independent one was TFD. TFD also worked best in both prognostic models; the reliability of the models was substantially decreased if TFD was replaced by any other tumour-related markers. TFD is, however, not our invention. Several authors reported that the risk of LN involvement and poor survival is negatively associated with the thickness of the remaining fibromuscular cervical stroma around the tumour. Tsukamoto et al.^29^ in 1966 and Noguchi et al.^30^ in 1983 reported a 5-year survival rate of 100% in patients in whom the remaining normal stroma measured more than 5 mm in thickness. In 1987, Kishi et al.^31^ reported a low risk of LN positivity (7%) and high 5-year survival (92%) in patients with uninvolved stroma ≥3 mm, while corresponding figures were 37 and 26% if TFD was ≤3 mm. Landoni et al.^32^ in 1995 reported an increased risk of parametrial invasion in patients with TFD *≤* 3 mm. In our study, TFD (*≤* 3.5 mm) surpassed all other traditional markers which are related to tumour size. We can hypothesise that the distance between tumour and parametria better corresponds with the risk of extrauterine tumour spread than the tumour size or depth of stromal invasion, which does not take into account the size of the cervix and tumour location in the cervix. TFD in this study showed an inverse relationship to the presence of positive LN, thus supporting this hypothesis. TFD was by far the best marker in both predictive models for the entire population and for the LN-negative subgroup. TFD can be easily assessed by pre-operative imaging (MRI or expert US), and cut-off value was established at 3.5 mm so the factor could be binarised in predictive models.

The main limitation of the study is its retrospective design, which, however, can be partially compensated by the inclusion of all consecutive (eligible) patients. The main strength is the fact that all prognostic markers were assessed by a standardised protocol for imaging and pathology. Moreover, the majority of patients underwent SLN biopsy analysed by an intensive ultrastaging protocol, which increased the sensitivity of LN staging, especially detection of low volume disease (MIC and ITC). In addition, the treatment strategy in our institutional cohort was not significantly changed during the study period.

In conclusion, our study confirmed that LN involvement is a significant traditional prognostic factor for predicting RR in early-stage cervical cancer. Out of markers related to tumour size, we identified a new prognostic marker, TFD, which correlates the best with the recurrence risk and can be easily assessed by pre-operative imaging. Prognostic models, combining TFD with LN status or tumour type in the entire population and in LN-negative patients, are easy to use in routine clinical practice and are able to identify the smallest possible group of patients with the highest risk of recurrence. Other traditional markers, such as LVSI or DSI, were less significant predictive factors and they did not improve prognostic models. Our models should be validated as indications for adjuvant treatment in future studies.

## Supplementary information

Supplementary material

## Data Availability

The datasets used and/or analysed during the current study are available from the corresponding author on reasonable request.
