# Supplementary material for: Tumour-free distance: a novel prognostic marker in patients with early-stage cervical cancer treated by primary surgery
Source: Br J Cancer. 2020 Dec 14;124(6):1121–9. doi: 10.1038/s41416-020-01204-w (PMC7961006; doi:10.1038/s41416-020-01204-w)
Supplement: Supplementary file 1 — Supplementary material [file 41416_2020_1204_MOESM1_ESM.docx]

**Supplementary material**

**Tumour-free distance: a novel prognostic marker in patients with early-stage cervical cancer treated by primary surgery**

David Cibula^1,^*, Jiri Slama^1^, Lukáš Dostálek^1^, Daniela Fischerová^1^, Anna Germanova^1^, Filip Frühauf^1^, Pavel Dundr^2^, Kristyna Nemejcova^2^, Jiri Jarkovsky^3^, Silvie Sebestova^5^, Andrea Burgetová^4^, Martina Borčinová^1^ and Roman Kocián^1^

^1^ Gynecologic Oncology Center, Department of Obstetrics and Gynecology, First Faculty of Medicine, Charles University in Prague and General University Hospital in Prague, Apolinarska 18, Prague 2, 12800, Czech Republic

^2^ Department of Pathology, First Faculty of Medicine, Charles University in Prague and General University Hospital in Prague, Studnickova 2, Prague 2, 12800, Czech Republic

^3^ Institute of Biostatistics and Analyses, Faculty of Medicine, Masaryk University, Kamenice 126/3, Brno, 62500, Czech Republic

^4^ Department of radiology, First Faculty of Medicine, Charles University in Prague and General University Hospital in Prague, U nemocnice 499/2, Prague 2, 12808, Czech Republic

^5^ Institute of Health Information and Statistics of the Czech Republic, Palackeho namesti 4, P.O. Box 60, Prague 2, 12801, Czech Republic

******* Correspondence: [dc@davidcibula.cz](mailto:dc@davidcibula.cz)

**Table S1**: Preoperative ultrasound checklist to scan cervical cancer

| ***ULTRASOUND CHECKLIST TO SCAN CERVICAL CANCER*** | |
| --- | --- |
| **Weight _______ kg, height __________ cm** |  |
| **Imaging quality** | good/moderate/poor |
| ***TRANSVAGINAL/TRANSRECTAL APPROACH*** | |
| **Position of the uterus** | anteverted / retroverted / upright position |
| **Size of the uterus** | CC___/AP___/LL___ (mm) |
| **Size of the uterine cervix** | CC___/AP___/LL___ (mm) |
| **Cervical tumour identification** | no/yes |
| **Size of tumour** | CC___/AP___/LL___ (mm) |
| **Cranial tumour-free margin for planning FST** | __________ (mm) |
| **Tumour echogenicity** | uniform /non-uniform |
| **Tumour echogenicity compared to adjacent cervical stroma** | hypoechogenic, isoechogenic, hyperechogenic |
| **Tumour colour score (subjective assessment)**  (1-no colour flow signals, 2-minimal colour, 3-moderate colour, 4-abundant colour) | 1–4 |
| **Tumour localisation** | endocervical / exocervical / combined |
| **Depth of stromal invasion (subjective assessment)** | ˂50%; 50% - 75%; ˃75%; the whole cervix |
| **Parametrial involvement**  (if absent, please continue to fill in TFD; if present, please continue to parametrial infiltration) | no/yes |
| **Minimum tumour-free distance (TFD)**  (the parameter is evaluated only if negative parametria and measured at the level of ventral, lateral and dorsal parametria attachment to the cervix) | ventral right__ /ventral left__ (mm)  lateral right__/lateral left __(mm)  dorsal right__/dorsal left___(mm) |
| **Parametrial involvement (Location, Grade, Size)**  Grade 1, disrupted peri-cervical ring  Grade 2, incipient infiltration of parametria ≤5mm  Grade 3, nodular infiltration  Grade 4, discontinual parametrial involvement;  size: width/length of parametrial infiltration | ventral parametria  rightgrade__, size: _/__(mm)  leftgrade __, size __/__ (mm)  lateral parametria  rightgrade__, size: _/__(mm)  leftgrade __, size __/__ (mm)  dorsal parametria  rightgrade__, size: _/__(mm)  leftgrade __, size __/__ (mm) |
| **Involvement of uterine body** | no/yes |
| **Vagina infiltration** | no/yes  ventral (up to 1/3, 2/3, the whole length of vagina)  dorsal (up to 1/3, 2/3, the whole length of vagina) |
| **Infiltration of urinary bladder**  Grade 1, infiltration of the echogenic outer layer  Grade 2, disruption of hypoechogenic muscle layer  Grade 3, disruption of all layers with intraluminal tumour spread | no/yes (grade) |
| **Infiltration of rectum**  Grade 1, infiltration of the echogenic outer layer  Grade 2, disruption of hypoechogenic muscle layer  Grade 3, disruption of all layers with intraluminal tumour spread | no/yes (grade) |
| **Infiltration of ovaries** | no/yes (right/left) |
| **Pelvic peritoneal involvement** | no/yes |
| **Visceral lymph node/-s involvement**  (Location: ventral parametria right[1],ventral parametria left [2], lateral right [3], lateral left [4], dorsal (uterosacral ligaments) right [5],dorsal (uterosacral ligaments) left [6];  Grade: normal or reactive, suspicious, infiltrated | no/yes  location (1–6), grade, size in short axis [____, mm]) |
| **Para-iliac lymph node/-s involvement**  (Location: internal iliac right[1], external iliac right[2], common iliac right[3], internal iliac left[4], external iliac left [5], common iliac left [6];  Grade: reactive, suspicious, infiltrated | no/yes  location (1–6), grade, size in short axis [____, mm]) |
| **Free fluid in Pouch of Douglas** | _____________(mm) |
| ***ABDOMINAL APPROACH*** | |
| **Liver parenchyma** | homogeneous (yes/no)  heterogeneous (yes/no)  diffuse (specify)  focal lesion/-s (specify) |
| **Spleen** | homogeneous (yes/no)  heterogeneous (yes/no)  diffuse (specify)  focal lesion/-s (specify) |
| **Hydroureter or hydronephrosis**  Grade 1, dilation of renal sinus  Grade 2, dilatation of renal sinus and calyces  Grade 3, sacciform dilation of renal sinus and calyces with renal parenchyma atrophy | no/yes (grade) |
| **Paraaortic lymph node/-s involvement**  (location: inframesenteric [1], supramesenteric [2]; Grade: reactive, suspicious, infiltrated) | no/yes  location (1, 2), grade, size in short axis [____, mm]) |
| **Visceral lymph node/-s (around visceral branches of aorta) involvement**  (location: celiac [1], around visceral branches of coeliac trunk, mesenteric [2], around visceral branches of mesenteric arteries; grade: reactive, suspicious, infiltrated) | no/yes  location (1, 2), grade, size in short axis [____, mm]) |
| **Peripheral lymph node/-s involvement**  **(location: inguino-femoral lymph nodes right** superomedial [1], superolateral [1], superficial distal [3], deep[4]; **inguino-femoral lymph nodes left** superomedial [5], superolateral [6], superficialdistal [7], deep [8]; **scalene (supraclavicular) lymph nodes left** [9]; **Grade:** reactive, suspicious, infiltrated) | no/yes  location (1-9), grade, size in short axis [____, mm]) |
| **Ascites** | no/yes |
| ***Conclusion*** |  |
| Preoperative staging T____ N____ M____.  ***Other comment:*** | |

CC: craniocaudal; AP: anteroposterior; LL: laterolateral diameter; FST: fertility-sparing treatment.

**Table S2**: Detailed description of the hysterectomy specimen processing

| Tumour size | If the tumour was not macroscopically visible (e.g. due to the previous surgery), the exocervix was processed in a manner similar to cone biopsy specimen. If the tumour was large and macroscopically visible, only selected areas were sampled, allowing assessment of maximal depth of stromal invasion (DSI), surgical margin evaluation, spread of the tumour, and its relation to the adjacent structures. Subsequent sections of the endocervix were processed in histotopograms (whole mount sections) together with a part of the adjacent parametria. The remaining parts of the parametria were dissected from the uterus and processed in longitudinal sections. |
| --- | --- |
|  | Each tumour was measured in three dimensions (two determining the horizontal size, one depth of the invasion). We used a combination of macroscopic and microscopic measurements to determine maximal tumour dimensions. |
|  | If a previous procedure (e.g. cone biopsy, etc.) was performed, the evaluation of the overall size also took into account the results of the previous biopsy examination. When we determined the maximum horizontal dimension, we counted the horizontal dimensions in both samples (with a certain risk that the horizontal dimension would be overestimated). |
| Depth of invasion | Similarly as for the tumour size, in determining the depth of invasion, we counted the maximum depth of invasion in both samples. |
|  | Horizontally, the tumour was measured in two dimensions (longitudinal, circumferential). One horizontal dimension (longitudinal) was determined from the section with the largest extent of the invasion. If the invasion was present in only one block, the second horizontal dimension (circumferential) was determined as a block width (usually 2–3 mm) affected by the tumour. If the invasion was present in multiple blocks, then the second horizontal dimension was determined as the estimated block width multiplied by the number of blocks with invasive tumour. When a single invasive focus was present in the section, its largest horizontal dimension (longitudinal) was measured. If there were multiple small clustered invasive foci originating from one crypt or surface epithelium area, the measurements included all these foci (measured from the edge of the first focus through the entire horizontal dimension to the distal edge of the last focus). If there were several separate invasion foci in one excision, the measurements included all these foci (measured from the edge of the first focus through the entire horizontal dimension to the distal edge of the last focus), until these areas were interrupted by at least 2 mm of uninvolved cervical tissue with non-dysplastic epithelium. In this case, we regarded the lesions as multifocal carcinomas. The depth of the invasion was measured from the basal epithelial membrane (surface or crypt) from which invasive carcinoma had penetrated to the deepest point of the invasion. If the invasive focus (or foci) was associated with the dysplastic epithelium from which it originated, it was measured from this epithelium. If the invasive focus (or foci) was not associated with a dysplastic epithelium, it was measured from the basal membrane of the closest dysplastic epithelium (surface or crypt). If the dysplastic epithelium from which the invasive focus (or foci) was not present, the depth of invasion was measured from the basal membrane of the nearest surface epithelium, regardless of whether dysplasia was present. |

**Table S3**: Sites of recurrence and type of adjuvant treatment according to lymph node status and administration of adjuvant treatment

|  | Localization and number of recurrences | | Type of adjuvant treatment |
| --- | --- | --- | --- |
| N0 patients with adjuvant treatment (N=33) | Multiple | 1 | 1xRT |
|  | Isolated pelvic | 2 | 1xRT, 1xCHRT |
|  | Unknown | 2 | 1xRT, 1xCHRT |
|  | No recurrence | 28 | 23xRT, 5xCHRT |
| N0 patients without adjuvant treatment (N=287) | Multiple | 5 |  |
|  | Isolated pelvic | 10 |  |
|  | Isolated distant | 1 |  |
|  | Unknown | 2 |  |
|  | No recurrence | 269 |  |
| N1 patients with adjuvant treatment (N=42) | Multiple | 10 | 4xRT, 6xCHRT |
|  | Isolated distant | 1 | 1xRT |
|  | Unknown | 6 | 1xRT, 5xCHRT |
|  | No recurrence | 25 | 8xRT, 17xCHRT |
| N1 patients without adjuvant treatment (N=17) | Multiple | 1 |  |
|  | Isolated pelvic | 1 |  |
|  | Unknown | 1 |  |
|  | No recurrence | 14 |  |

CHRT: chemoradiotherapy; RT: combined radiotherapy
